# The Current State of Clinical Trials Studying Hydrocephalus: An Analysis of ClinicalTrials.gov

**DOI:** 10.7759/cureus.10029

**Published:** 2020-08-25

**Authors:** Mickey E Abraham, Roman Povolotskiy, Justin Gold, Max Ward, Julian L Gendreau, Antonios Mammis

**Affiliations:** 1 Department of Neurosurgery, University of California, San Diego, USA; 2 Department of Neurosurgery, Rutgers New Jersey Medical School, Newark, USA; 3 Graduate Medical Education, Eisenhower Army Medical Center, Fort Gordon, Augusta, USA

**Keywords:** review of clinical trials, post-hemorrhagic hydrocephalus, pediatric hydrocephalus, normal pressure hydrocephalus, hydrocephalus

## Abstract

Introduction

Hydrocephalus is a significant public health concern estimated to affect 380,000 new individuals annually. In addition, it exhibits an increasingly high financial burden for the healthcare industry. Clinical trials are the gold standard for evaluating preventative and therapeutic strategies to bring potential treatments to the forefront of clinical practice.

Methods

A study of the ClinicalTrials.gov was conducted in April 2019 to examine all current and previously reported clinical trials studying hydrocephalus. Studies were reviewed to extrapolate information to characterize the current state of research being conducted for hydrocephalus.

Results

In total, 80 clinical trials met inclusion criteria and were analyzed: 48.8% were observation and 51.2% were interventional. Of those, 55% have been completed while 30.0% are still recruiting, and 15.0% are not yet recruiting. The United States has the most clinical trials (42.0%) and a plurality of trials has a sample size of 0-50 participants. The majority of studies included only adults (53.8%). Of those studies, 54.0% were cohort and the majority were prospective (74.0%). Of the different types of hydrocephalus, normal pressure hydrocephalus and pediatric hydrocephalus have generated the most interest for research comprising a majority of the clinical trial registry. While 44 of the trials are complete, only 20 have published results in peer-reviewed literature highlighting the need for improvement in publishing study results even if the results of the trials are null.

Conclusion

Most clinical trials to date have pertained to the treatment of normal pressure hydrocephalus and pediatric hydrocephalus. While great advancements have been made for the treatment of hydrocephalus, there remains much room for improvements in therapeutic interventional modalities as well as ensuring the reporting of all undertaken clinical trials.

## Introduction

Hydrocephalus, first described by Hippocrates as early as the fifth century BCE, is an abnormal accumulation of cerebrospinal fluid (CSF) within the ventricles of the brain either due to insufficient CSF reabsorption or CSF overproduction [1]. It is a significant public health concern estimated to affect 380,000 new individuals annually [2]. As a result, CSF diversion procedures such as ventriculoperitoneal shunting (VPS) and endoscopic third ventriculostomy (ETV) accrue a total cost of $2 billion nationwide [3]. One study suggests that treating hydrocephalus in the elderly, has the potential to lower five-year Medicare expenditures by approximately $184.3 million in the United States [4].

Hydrocephalus can develop for a variety of reasons. Obstructive, or noncommunicating, hydrocephalus occurs when CSF does not flow properly between ventricles or out of the ventricles due to an obstruction. This obstruction can be the result of congenital malformations or ventricular narrowing due to other pathologies such as cancer or trauma [5]. Nonobstructive, or communicating, hydrocephalus occurs when CSF flows out of the ventricles and into the spinal canal, but the tissue surrounding the brain and spinal cord is unable to reabsorb it adequately enough to prevent excess accumulation of the fluid [6]. Major types of hydrocephalus include congenital hydrocephalus, acquired hydrocephalus, and normal pressure hydrocephalus (NPH) [7].

In 2012, researchers from across the globe convened at the symposium, "Opportunities in Hydrocephalus Research: Pathways to Better Outcomes," ultimately recommending four areas of hydrocephalus research to most efficiently improve patient care [8]. These include: discovering genetic and pathophysiologic causes of the diagnosis, developing improved biomarkers for diagnosis, discovering new bioengineering and surgical advances for treatment, and also enhancing neuropsychological and quality of life initiatives. These comprehensive recommendations accentuate most of the ongoing research of hydrocephalus and have the potential to refine diagnoses, improve clinical outcomes, and reduce healthcare expenditure [9,10].

Clinical trials remain the gold standard for evaluating novel diagnostic, preventative, and therapeutic strategies before it reaches the forefront of clinical practice [11]. Therefore, the present study examined a major worldwide registry, ClinicalTrials.gov, for all trials involving hydrocephalus. This analysis identified and characterized the current state of clinical trials focused on hydrocephalus. This analysis attempts to understand what steps have already been taken, and what areas of research remain to be explored in order to continue improving the management of hydrocephalus.

## Materials and methods

ClinicalTrials.gov is a public trial registry provided by the United States National Library of Medicine and the United States Food and Drug Administration that contains over 301,795 research studies conducted in 208 countries and all 50 states. The authors conducted a search on April 8, 2019 to examine all current clinical trials studying hydrocephalus.

The trials were obtained from the ClinicalTrials.gov website using the advanced search function for the search term “hydrocephalus” under “condition.” Of those identified by this search criteria, clinical trials were excluded from the analysis if they were suspended, terminated, withdrawn, or had unknown status. For the trials that met inclusion criteria, the following information was used in the final analysis: registered identifier number, official title, recruitment status, study type, primary purpose, year of initiation, intervention, country, study type, sample size, year of initiation, year of completion, time perspective, primary purpose, intervention, participant age, country of origin, and primary outcome. In addition, an effort was made to retrieve the results of completed trials for further analysis. For all completed trials, an online Medline database search for published results was conducted on May 2, 2019, using each clinical trial's registered identifier number. Descriptive statistics were used to analyze the variables. 

## Results

In total, 123 trials were identified. Of these identified, 43 trials were excluded from the analysis due to suspended, terminated, withdrawn, or unknown status. The remaining 80 studies were included in this study. A complete list of included studies can be found in Appendix 1. 

**Figure 1:**
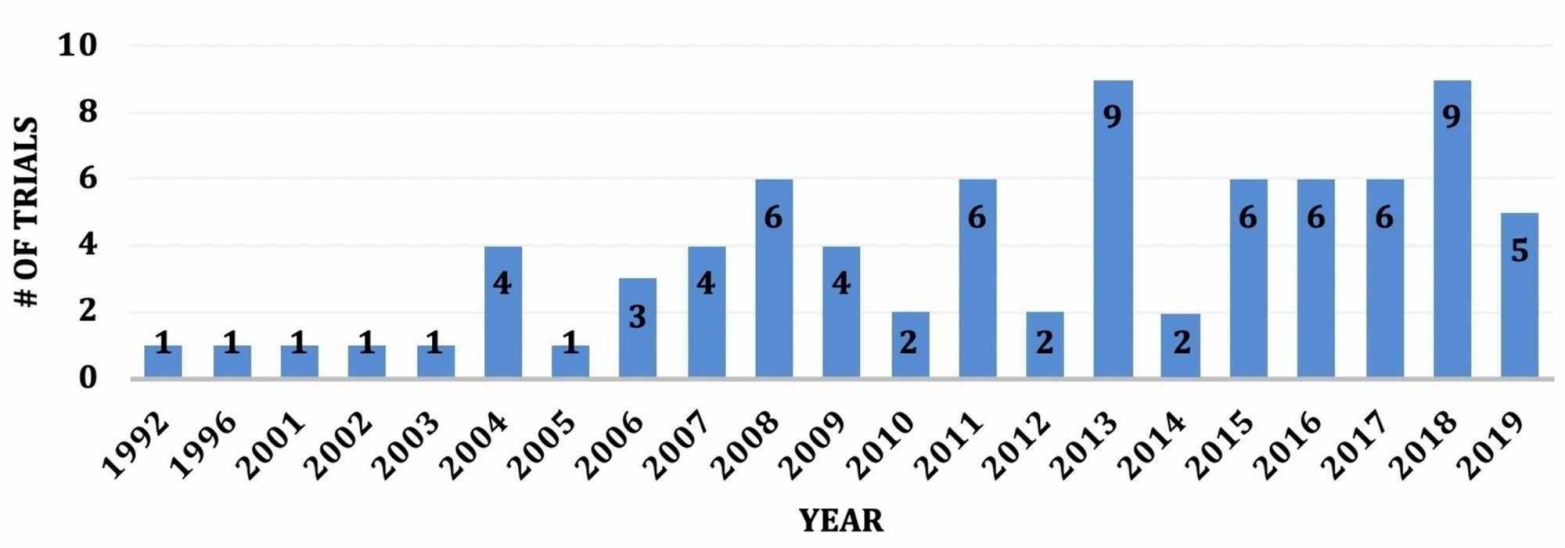
Number of Clinical Trials Beginning Stratified by Year

Among the 80 eligible trials, 39 (48.8%) were observational and 41 were interventional (51.2%). The trials took place from 1992 to 2019, with 43 (53.8%) studies beginning after 2013. Therefore, there appears to be an increasing trend of clinical trials since 1992 (Figure 1). The majority of trials, 44 (55.0%), have been completed, 24 (30.0%) are still recruiting, and the remaining 12 (15.0%) are not yet recruiting. Of the 44 completed trials, the average time to completion was 50 months. 

The United States of America has the most clinical trials, 45 (42.0%), with the rest originating in predominantly westernized or European countries (Table 1). Enrollment in these trials included a wide range: 30 (37.5%) trials had a sample size of 0-50, 21 (26.3%) had a sample size of 50-100, 25 (31.3%) had a sample size of 101-500, and 4 (5.0%) had an enrollment that exceeded 501. The median number of participants was 158 among all trials. When stratified by participant age, 43 (53.8%) included only adults (18 years and older), 18 (22.5%) included only children (aged 0-18 years), and 19 (23.8%) included all ages. 

**Table 1:** Nationality of Registered Clinical Trials for Hydrocephalus

Country	N	%
Austria	1	1.3%
China	3	3.8%
Denmark	1	1.3%
France	6	7.5%
Germany	3	3.8%
Israel	2	2.5%
Japan	1	1.3%
Malaysia	1	1.3%
Netherlands	1	1.3%
Spain	1	1.3%
Sweden	5	6.3%
Switzerland	4	5.0%
Turkey	1	1.3%
Uganda	1	1.3%
United Kingdom	1	1.3%
USA	37	46.3%
Multiple Countries	10	12.5%
N/A	1	1.3%

Of the 39 observational studies, the majority, 21 (54.0%), were cohort, while the rest were case-control (4, 10.2%), case-only (8, 20.5%), or unspecified (6, 15.4%). The study type was diverse as 29 (74.0%) were prospective, 7 (18.0%) were cross-sectional, 2 (5.0%) were retrospective, and 1 (2.5%) was not available. A majority of the clinical trials lasted for one to five years (Table 2).

**Table 2:** Number of Clinical Trials Stratified by Study Length

Duration	N	%
< 1 year	8	10.0%
1-5 years	35	43.8%
> 5 years	9	11.3%
Ongoing	28	35.0%

Of all clinical trials, 28 (35%) are studying NPH. Of these studies, eight are studying the effectiveness of VPS to improve current techniques, four focus on improving current imaging techniques for diagnosis, three are studying CSF biomarkers for diagnosis, two compare the effects of ETV to VPS for treatment, two are studying cognitive and development outcomes, and two are studying CSF dynamics to optimize treatment. One robust study is focused on building a registry, studying vascular risk factors, improving patient education, elucidating potential drug targets, improving gait treatments, improving treatments of urinary symptoms, and improving choroid plexus cauterization.

Of all clinical trials, 25 (31.3%) are studying pediatric hydrocephalus. Of these studies, six are studying improving shunt function, six focus on improving current imaging modalities and advancing novel imaging techniques for diagnosis, five compare the effects of ETV to VPS for treatment, two provide data for registries to support hypothesis generation and study design development for clinical trials, two are studying cognitive and development outcomes, two are testing potential alternative anesthetics for obtaining MRI in this challenging population, one is studying CSF biomarkers to assess the severity of disease and response to treatment, and one is studying head circumference of hydrocephalic Ehlers-Danlos patients who develop dysautonomia later in life. 

Of all clinical trials, four (5.0%) are studying post-hemorrhagic hydrocephalus. These trials are examining whether early use of lumbar puncture leads to less shunt surgery, assessing the optimal conditions for EVD cessation, determining the role of proteomics in cerebral vasospasm following SAH, and evaluating hydrocephalus as a predictor of functional disability and quality of life.

The remaining 23 (28.8%) trials study all forms of hydrocephalus. Ten trials are examining the effectiveness of VPS and improving current techniques for treatment, five are focused on improving current imaging techniques for diagnosis, two are studying alternative methods to measure intracranial pressure, two are investigating CSF flow dynamics, and one study each is focused on patient education, prevention, and improving cognitive function.

To date, only four of the 44 completed trials have posted a statistical analysis on ClincalTrials.gov. Efforts were made to obtain the results of the other 40 trials and a search of the Medline database demonstrated that 20 of the studies had published results in peer-reviewed literature. 

## Discussion

Advancements in the diagnosis and treatment of hydrocephalus have rapidly improved over the last century [12]. From the first attempt to create a permanent ventriculo-subarachnoid-subgaleal CSF diversion by Mikulicz in 1893, to the first functional valve implantation devised by Nulsen and Spitz in 1949, the basis of treatment for hydrocephalus has largely remained the same [13,14]. In spite of several innovations and technical modifications, shunts, which have evolved and matured to become the standard of care for all types of hydrocephalus, are not without complications [15]. Further, since shunts remain a source of lifetime concern for the patient, parents, and family, the desire for shunt freedom has led to a resurgence of ETV techniques and other endeavors that aim to change the current standard [16]. Of the different types of hydrocephalus, NPH and pediatric hydrocephalus have generated the most research interest and comprise a majority of the current clinical trial registry.

There has been a sharp increase in the number of trials being conducted since 2013, which holds promise for future trends in hydrocephalus research and clinical management. This study found that the clinical trials were almost evenly split into interventional and observational studies. This demonstrates that while great advancements have been made for the treatment of hydrocephalus, there remains much room for improvements in current interventions due to the low number of interventional clinical trials. While more than half of the trials are reported as complete, only four have results available on ClinicalTrials.gov and only 20 of these have published their results in peer-reviewed literature. Therefore, there should be an encouragement for the neurosurgical community to publish results of all clinical trials, even if the findings are null or negative.

NPH is the most common form of hydrocephalus in adults, and it is estimated that over 700,000 people in the United States have NPH. It is characterized by dilated cerebral ventricles and it presents with a clinical trial involving impaired gait, cognition, and urinary control [17]. It is estimated that over 700,000 people in the United States have NPH [18]. Pediatric hydrocephalus has a prevalence of approximately 6 in 10,000 live births, with a neonatal mortality rate of 13% before hospital discharge [19]. According to nationally representative data from 2008, pediatric hydrocephalus accounts for 38,200 to 39,900 hospital admissions, 391,000 to 433,000 hospital days, and $1.4 to $2.0 billion in total hospital charges annually in the United States [20]. Because NPH and pediatric hydrocephalus are both prevalent diseases with a high cost of care, the completion and publication of the ongoing research will help clarify causes, hasten diagnosis, improve treatment, and increase the quality of life. 

NPH is difficult to diagnose because of its insidious onset and nonspecific symptomatology. However, it is one of only a few reversible causes of dementia, making early diagnosis critical. The published results of clinical trials studying NPH demonstrate that much remains to be learned from this complex pathology and that NPH is a multi-etiological clinical entity, possibly overlapping pathophysiologically with cerebrovascular disease and Alzheimer's disease [21]. A high level of clinical suspicion is necessary to piece together the clinical picture and ultimately make the diagnosis. MRI is a useful modality because it allows for MR phase imaging of CSF flow which provides pathophysiological information of potential clinical importance [22]. Additionally, new neuropsychological tests have been developed to assess cognitive impairment, which can assist in early diagnosis and improve outcomes [23].

Current evidence-based guidelines and best practices for hydrocephalus treatment suggest that shunts are superior to ETV in infants [24]. Regarding imaging, the degree of fetal ventriculomegaly based on the ultrasound and MRI measurements had a predictive value for successful live birth while ultrasound-guided shunt insertion was unable to consistently place catheters into the frontal horn of the ventricles. However, laparoscopically guided distal VPS placement provides definite patient benefit by allowing shunt placement under direct vision, all while allowing for reduced trauma to the abdominal wall and avoiding intra-abdominal adhesions [25-27]. 

A majority of the clinical trials of hydrocephalus included in the database have begun over the last five years. Since the average time to completion of the completed trials was 50 months, we can expect a host of new results to become available over the next few years. Practitioners should anticipate the publication of these results and look for practice-changing recommendations. 

Limitations

First, while ClinicalTrials.gov accounts for more than 80% of all studies in the World Health Organization’s International Clinical Trials Registry platform, it does not include all clinical trials [28]. Second, this study only analyzes the characteristics of the registered trials but does not assess the quality of the research being conducted. As more data become available, the quality of the research will have to be further scrutinized. 

## Conclusions

The current study provides an overview of registered trials on ClinicalTrials.gov studying hydrocephalus. Approximately half of the trials are interventional and half are observational. A majority of the trials are studying NPH and pediatric hydrocephalus. While 44 trials are complete, only 20 have published results in peer review highlighting the need to translate these results into peer-reviewed publications for the edification of the neurosurgical community. Doing so will only serve to further the growing trend in hydrocephalus research, offering hope to patients and their families.

## Appendices

**Table 3:** List of Clinical Trials Included in the Final Analysis CSF: cerebrospinal fluid, VPS: ventriculoperitoneal shunting, ETV: endoscopic third ventriculostomy, NPH: normal pressure hydrocephalus, TVH: triventricular hydrocephalus, ETV/CPC: endoscopic third ventriculostomy with choroid plexus cauterization, SED: smart external drain, HIFE: high intensive functional exercise, PAL: pacifier-activated-lullaby, BSID-III: Bayley scales of infant development, third edition, TUG: timed up and go, IHC: immunohistochemical, ICP: intracranial pressure, INPH: idiopathic normal pressure hydrocephalus, VCL: ventricular catheter location, HCRN: Hydrocephalus Clinical Research Network.

ClinicalTrials.gov Identifier	Official title	Type of Hydrocephalus	Country	Intervention (if applicable)	Primary outcome
NCT01936272	Neurocognitive Outcomes and Changes in Brain and Cerebral Spinal Fluid (CSF) Volume After Treatment of Post-Infectious Hydrocephalus (PIH) in Ugandan Infants by Shunting Versus ETV/CPC	Neonatal postinfectious hydrocephalus	Uganda	Chhabra shunt placement	Change of age-normed BSID-III scores
NCT01909960	Benefit of Flow Magnetic Resonance Imaging in the Management of Normal Pressure Hydrocephalus	NPH	France	Flow imaging	CSF stroke volume evolution other six months
NCT02230124	Magnetic Resonance Elastography in Hydrocephalus	Neonatal postinfectious hydrocephalus	USA	MRE (magnetic resonance elastography)	Mean shear modulus
NCT02425761	A Randomized Controlled Trial of Anterior Versus Posterior Entry Site for Cerebrospinal Fluid Shunt Insertion	Pediatric hydrocephalus	USA, Canada	Ventriculoperitoneal shunt insertion surgery	Shunt failure determined by clinical and radiographic findings
NCT01797627	Ventricular Size Involvement in Neuropsychological Outcomes in Pediatric Hydrocephalus (VINOH)	Congenital hydrocephalus	USA, Canada		Association between ventricle size and neuropsychological outcome
NCT01041950	Lumbar Drainage for Communicating Hydrocephalus After Intraventricular Hemorrhage: A Randomised, Controlled Trial (LUCAS-IVH: LUmbar CAtheter for Severe IntraVentricular Hemorrhage)	Post-hemorrhagic hydrocephalus	Germany	Lumbar drainage	The requirement of permanent VPS
NCT01798641	Efficacy of Shunt Surgery in Normal Pressure Hydrocephalus: A Randomized Cross-Over Study	NPH	USA	MIETHKE proGAV®/MIETHKE proSA® (Braun, Tuttlingen, Germany)	1. Tinetti score on open shunt vs. closed shunt 2. TUG score on open shunt vs. closed shunt 3. Medical College of Virginia (MCV) Gait Grade on open shunt vs. closed shunt 4. Kiefer score on open shunt vs. closed shunt 5. Kubo score on open shunt vs. closed shunt
NCT02651337	Quantitative Characterization of Safe Irrigation for Ventricular Shunt Catheters	All	USA	Alivio in line flusher	Characterization of safe shunt catheter "flushing" using a quantitative assessment of saline injection volume (mls) required to flush the CSF shunt using the Alivio in-line Flusher
NCT00286104	The Impact of Ventricular Catheter Impregnated With Antimicrobial Agents on Infection in Patients With Ventricular Catheter: A Prospective Randomized Study	All	China	Antibiotics-impregnated ventricular catheter (Bactiseal®, Codman, Johnson & Johnson, Raynham, USA)	1. CSF infection rate 2. Extracranial infection rate
NCT02575105	Bispectral Index Monitoring of Propofol Anesthesia in Patients With Hydrocephalus: A Prospective Observational Study	Congenital hydrocephalus	Austria	Bispectral index value	The difference in bispectral index value
NCT01815775	Predictive Value of Flow MRI in Normal Pressure Hydrocephalus Surgery	NPH	France	Clinical and imaging examinations	CSF stroke volume
NCT00196196	A Precision and Accuracy Study of the Codman Valve Position Verification (VPV) System	All	USA	Codman VPV system	Percentage of participants who achieved "adjustment complete" and a consensus X-ray reading
NCT01265251	Evaluation of a Computerised Neuropsychological Test Battery for Idiopathic Normal Pressure Hydrocephalus (INPH)	NPH	Sweden	Computerized neuropsychological test	1. Validity; 2. reliability; 3. feasibility
NCT00600795	Prognostic Value of Transforming Growth Factor-Beta 1 in Normal Pressure Hydrocephalus	NPH	USA	CSF collection	1. TGF beta-1 levels 2. Mini-mental status exam 3. Modified Barthel index 4. Tinetti mobility assessment
NCT01801267	A Randomized Controlled Trial of Endoscopic Third Ventriculostomy Versus Ventricular Shunt for Children With Communicating Hydrocephalus	Communicating hydrocephalus	USA	Endoscopic third ventriculostomy	Time until the need for further CSF-related surgeries
NCT00652470	International Infant Hydrocephalus Study: A Multicentre, Prospective Study	TVH	USA, Argentina, Brazil, Canada, Germany, Hungary, India, Israel, Italy, Netherlands, Poland, Russia, Serbia, Spain, Turkey, UK	Endoscopic third ventriculostomy	Health status outcome as measured by the Health Utilities Index
NCT01053312	A Principal, Open-Label, Single Center Study to Validate the Detection of Cerebral Cortical Amyloid With Flutemetamol (18F) Injection in Subjects Previously Biopsied	NPH	USA	Flutemetamol	1. Quantitative estimates of brain uptake [18F] Flutemetamol and the quantitative IHC estimates of amyloid levels in biopsy samples previously obtained 2. Comparison between brain uptake of [18F] Flutemetamol amyloid level from IHC assay and a stained biopsy tissue specimen
NCT03595033	iPad Application Based Therapy Intervention in School Age Children With Surgically Treated Hydrocephalus	Pediatric hydrocephalus	USA	iPad app-based intervention	1. General intellectual functioning, including visual-spatial reasoning 2. Basic visual perception, visual-motor integration, and graphomotor skills 3. Visual-spatial processing 4. Visual-spatial processing and mental rotation 5. Selective visual attention 6. Visual-spatial decision making and visual-motor speed 7. Fine motor dexterity 8. Visual-motor control 9. Neuroanatomical alterations in brain tissue structure
NCT01115270	A Study Comparing Shunt Placement Versus Endoscopic Third Ventriculostomy in the Treatment of Hydrocephalus	NPH	USA	Non-invasive measures	Intracranial compliance
NCT00743457	Pediatric Ocular Ultrasound for VPS Failure	Pediatric hydrocephalus	USA	Ocular ultrasound	1. Optic nerve sheath diameter 2. Clinical VPS failure
NCT02016352	Cerebrospinal Fluid Proteome in Hydrocephalus	NPH	France	patient CSF extraction with hydrocephalus	CSF proteome measure
NCT01323764	A Double-Blinded Comparison of the Accuracy of ShuntCheck, a Non-Invasive Device, to Radionuclide Shunt Patency Test in Evaluating Shunt Function in Patients With Adult Hydrocephalus With Possible Shunt Obstruction	NPH	USA	Shunt check test	Sensitivity and specificity of ShuntCheck vs SPS
NCT03113799	A Study to Evaluate the Performance and Safety of Aqueduct's Smart External Device (SED) Compared to Standard EVD Drains	All	USA	Smart external drain	Staff interactions
NCT01811589	Randomized Controlled Multi-Center Trial Comparing the Ventricular Catheter Location Between Instrument Guided and Freehand Placement	All	Germany	Thomale-guide	Rate of the primary successful ventricular catheter placement with a grade I or grade Ib and location in the ipsilateral ventricle
NCT01976559	Comparison of Continuous Noninvasive and Invasive Intracranial Pressure Measurement - Celda Infusion Subprotocol	All	Sweden	Tympanic membrane displacement	Noninvasive ICP
NCT02325583	Baskent University Institutional Review Board	All	Turkey		Number of newborns provided qualitative images for MRI following oral glucose administration
NCT00182390	A Randomized Controlled Trial of Two Hemoglobin Thresholds for Transfusion in Newborns <1000 g Birth Weight	All	USA		1. Combined mortality or survival to tertiary hospital discharge without severe morbidity 2. Combined mortality or survival with neurodevelopmental disability (non-ambulatory cerebral palsy, blindness, deafness, and cognitive delay)
NCT01799018	Role of Proteomics and Metallomics in Cerebral Vasospasm Following Subarachnoid Hemorrhage	All	USA		1. Protein identification and concentration 2. Metal ion identification and concentration
NCT00280904	A Registry for Comparing Catheter-Related Infection Rates Among Various Shunt Systems in the Treatment of Hydrocephalus	All	USA, Canada, China, Singapore, India		Number of subjects with shunt infections
NCT01885468	X-Ray Verified Accuracy of the Aesculap - Miethke proGAV Adjustable Shunt Pressure Setting Verification Instrument	All	USA		Level of agreement between the proGAV® verification instrument measurement and X-ray control measurements
NCT02381977	Prevalence of Acute Critical Neurological Disease in Children: A Global Epidemiological Assessment	All	USA		Prevalence of acute brain insult
NCT00870675	MRI of Ventriculomegaly: Morphology and Outcome	Fetal ventriculomegaly	USA		1. Postnatal imaging abnormalities 2. Postnatal developmental delay
NCT00221091	Clinical Study of Idiopathic Normal Pressure Hydrocephalus for Neurological Improvement	NPH	Japan		Modified Rankin scale score
NCT00233701	Normal Pressure Hydrocephalus (NPH) Registry	NPH	USA		Database to describe the population of patients with NPH presenting for treatment
NCT01374048	Are Intracranial Pressure Waves Measurable Through Lumbar Puncture?	NPH	No contacts or locations provided		Not available
NCT00001327	Establishing the Physiology of Syringomyelia	NPH	USA		Not available
NCT01319136	Novel Infusion Method for Describing CSF Dynamics	NPH	Sweden		Not available
NCT01570257	A Randomized Trial of High and Low Pressure Level Settings on a Programmable Ventriculoperitoneal Shunt Valve for Idiopathic Normal Pressure Hydrocephalus: Results of the Dutch Evaluation Program Strata Shunt (DEPSS) Trial	NPH	Netherlands		The number of subdural effusions, detected on CT scan, in patients showing clinical improvement after implantation of a ventriculoperitoneal shunt, at the end of the study
NCT01850914	Vascular Risk Factors, Subclinical and Manifest Vascular Disease in Patients With Idiopathic Normal Pressure Hydrocephalus	Pediatric hydrocephalus	Sweden		Differences in blood pressure between INPH-patients and sex- and age-matched community-based population
NCT01739179	Laparoscopically Assisted Ventriculoperitoneal Shunt Placement: A Prospective, Randomized Two-Arm Study	Pediatric hydrocephalus	Switzerland		The number of patients with overall shunt failure
NCT00652197	A Flow Monitor for Pediatric Hydrocephalic Shunts - Flow Sensor Study	Pediatric hydrocephalus	USA		Volumetric flow of patient CSF through an extra-ventricular drainage system
NCT01007786	The Ventricular Catheter Placement Study: Assessment of Efficacy and Safety of an Ultrasound Guided Shunt Insertion Technique	Pediatric hydrocephalus	USA, Canada		The primary endpoint for the study is VCL as assessed on the first post-operative scan (US, CT, or MRI brain)
NCT01556178	Collection of Blood and Cerebrospinal Fluid for Pediatric Brain Tumor Research	Pediatric hydrocephalus	USA		Levels of miRNAs in the blood and CSF.
NCT01367977	Head Circumference Growth in Children With Ehlers-Danlos Syndrome Who Develop Dysautonomia ("POTS" - Postural Orthostatic Tachycardia Syndrome) Later in Life - A Retrospective Analysis	Pediatric hydrocephalus	USA		Measurement of head circumference in Ehlers-Danlos patients (retrospectively), between the ages of birth and 15 months of age
NCT00692744	Study of Quality of Life After Aneurysmal Subarachnoid Hemorrhage in Patients Aged 70 Years or Older	Post-hemorrhagic hydrocephalus	France		Modified Rankin scale score
NCT01480349	Shunt Outcomes of Post-Hemorrhagic Hydrocephalus: A Network Pilot Study	Post-hemorrhagic hydrocephalus	USA, Canada		The proportion of temporization, conversion proportion, and surgery checklist scores
NCT00747682	Alterations in Cerebral Perfusion, Oxygenation, Electrical Activity, and Markers of Cerebral Damage Associated With Cerebro-Spinal Fluid Reservior Aspiration in Neonates With Post Hemorrhagic Hydrocephalus	Post-hemorrhagic hydrocephalus	USA		Determine if decreasing ventricular volume improves middle cerebral artery flow, cerebral oxygenation, and cortical neuronal electrical activity
NCT01108965	A Flow Monitor for Pediatric Hydrocephalic Shunts	Post-hemorrhagic hydrocephalus	USA		The volumetric flow of patient cerebrospinal fluid through an extra-ventricular drainage system
NCT00875758	Phase II Study of Late Versus Early Treatment of Post-hemorrhagic Ventricular Dilation in Preterm Infants	Post-hemorrhagic hydrocephalus	USA		Ventriculoperitoneal shunt-dependence
NCT03076723	Using Cerebrospinal Fluid Dynamics to Identify Shunt Responders in Idiopathic Normal Pressure Hydrocephalus and to Optimize Postoperative Clinical Improvement While Minimizing Overdrainage Related Complications - A Double Blind Randomized Study	NPH	Finland, Sweden	Simulated change in shunt opening pressure	1. Change in total score on the European INPH scale 2. Gait velocity 3. European INPH scale 4. Gait velocity
NCT02404740	Continuous Noninvasive Estimation of Intracranial Pressure to Assess Ventriculoperitoneal Shunt Malfunction in Patients With Hydrocephalus	All	USA		The presence or absence of ventriculoperitoneal shunt malfunction in patients with hydrocephalus
NCT03650101	Improving Infant Hydrocephalus Outcomes in Uganda: Predicting Developmental Outcomes and Identifying Patients at Risk for Early Treatment Failure After ETV/CPC	NPH	USA	ETV/CPC	1. BSID-III, cognitive-scaled score, the incidence of ETV/CPC treatment failure 2. Incidence of ETV/CPC treatment failure
NCT03269201	Mapping Functional Networks of Brain Activity (Brain Network Activation, BNA) Based on Analysis of Evoked Response Potential (ERP) EEG Signals in Patients With Movement Disorders	All	Israel		MDS-UPDRS part III score
NCT03828032	Alternations of Multi-Parameters Including Hemodynamic Concentration and Water Levels During Dehydration Therapy on Brain Edema Patients	All	China		1. Changes in oxyhemoglobin concentration 2. Water concentration
NCT03826056	Neurology Inpatient Clinical Education Trial	NPH	USA		Patient satisfaction scores
NCT03777774	Vacuum Drains vs Passive Drains vs no Drains in Decompressive Craniectomies - A Randomized Controlled Trial on Subgaleal Drain Complication Rates	Pediatric hydrocephalus	Malaysia		Subgaleal hematomas
NCT03779594	Acetazolamide for Treating NPH in Shunt-Candidates Patients: An Open Label Feasibility Trial	NPH	Israel	Acetazolamide	1. Change from baseline gait 2. Change from baseline balance
NCT03471702	A Secondary Study Evaluating Performance and Safety of Aqueduct's Smart External Drain (SED2)	Post-hemorrhagic hydrocephalus	USA	Aqueduct's SED	The number of subjects who require switching to a standard of care external drain
NCT02900222	Study of Endoscopic Choroid Plexus Cauterization for Adult Patients With Hydrocephalus and Risk Factors for Perioperative Complications Following Shunt Surgery.	NPH	USA	Endoscopic choroid plexus coagulation	The occurrence of post-operative complications
NCT03245138	Endoscopic Third Ventriculostomy Versus Ventriculo-Peritoneal Shunting in Idiopathic Normal Pressure Hydrocephalus	NPH	Germany	ETV	Kiefer index
NCT02495610	A Prospective Single-Centre Trial Investigating Novel Parameters for the Prediction of Ventriculoperitoneal Shunting Efficacy in Patients With Idiopathic Normal Pressure Hydrocephalus	NPH	Switzerland	Gait analysis and MRI	MRI
NCT02659111	Effects of Physical Training in Shunt-Operated Patients With Idiopathic Normal Pressure Hydrocephalus	NPH	Sweden	HIFE	1. Change in INPH-scale 2. Change in GAS
NCT03230032	RCT of Feeding Intervention With Pacifier Activated Device and Mother's Voice in Infants at High-risk for Cerebral Palsy	Congenital hydrocephalus	USA	Pacifier-activated device (PAL) system	Oral feeding efficiency
NCT03350750	A Placebo-Controlled Effectiveness in INPH Shunting (PENS) Trial: Proof of Concept	NPH	USA, Canada, Sweden	Programable CSF shunt valve	Gait velocity
NCT02408757	Multimodal Sonographic Monitoring of Cerebral Perfusion, Ventricle Seize and Optic Nerve Diameter During Weaning of Cerebrospinal Fluid Drainage Catheters: A Single Centre Observational Trial	All	Switzerland	Sonographic monitoring	Binary classification of patients in terms of change of width of CSF spaces by ultrasound
NCT03593330	Transitional Care Services: A Quality and Safety Process Improvement Programme in Neurosurgery	Pediatric hydrocephalus	UK	Transitional care program	Length of hospital stay
NCT03877107	Effect of Depletive Lumbar Puncture and Cerebrospinal Fluid Shunt Surgery on Lower Urinary Tract Dysfunction in Normal Pressure Hydrocephalus	NPH	France	Urinary symptoms profile questionnaire	1. Effect of depletive lumbar puncture on overactive bladder symptoms 2. Effect of depletive lumbar puncture on overactive bladder symptoms
NCT03092804	The Brain Structure and Neural Network Changing the Before and After Ventriculo-Peritoneal Shunting in the Normal Pressure Hydrocephalus Patients	NPH	China	Ventriculo-peritoneal shunting	1. The brain constructure and neural network changing one day after shunting 2. The brain constructure and neural network changing 90 days after shunting 3. The brain constructure and neural network changing one year after shunting
NCT03451669	Thermal Camera Detection of Ventriculoperitoneal Shunt Flow	All	USA		Determine if a smartphone-based thermal camera can determine flow in the distal tubing of a VPS
NCT03516162	Linking Day-to-day Digital Behaviour Captured on the Smartphone With Brain Function in Patients Undergoing Brain Surgery - The "Smart Surgery" Study	All	Switzerland		Change in pattern of smartphone-assessed day-to-day behaviour
NCT03531723	Brain Ultrasound in the Weaning of External Ventricular Leads	All	France		1. Inter-individual reproducibility of ultrasound measurement of the size of the third ventricle 2. Intra-individual reproducibility of ultrasound measurement of the size of the third ventricle 3. Intra-individual reproducibility of ultrasound measurement of the size of the third ventricle
NCT03382860	Estimation of Intracranial Pressure Using Non-Invasive Fundus Videos	All	Denmark		Absolute A/V ratio value to conventional intracranial pressure monitors
NCT02737163	Inadvertent Cerebral Spinal Fluid Valve Reprogramming: Prevalence and the Correlation With Signs, Symptoms, Radiographic Changes and the Exposure to Magnetic Fields.	All	USA		Frequency of inadvertent reprogramming of strata CSF shunt valves
NCT03698838	A Pilot, Prospective Study of Myelin Imaging Changes in Patients With Neurosurgical Diseases	All	USA		Myelin alterations read on MRI sequence
NCT03594136	Challenging Reference Values for Intracranial Pressure: A Prospective, Multicenter, Clinical Study	All	Czechia, Denmark		Normal intracranial pressure values
NCT03521518	Neuropsychometric Testing With Conner's Continual Performance Test-3 in Normal Pressure Hydrocephalus	NPH	USA		Attention and responsiveness
NCT03315637	Fetal Endoscopic Surgery for Spina Bifida	NPH	Spain		Capability to achieve successful closure of the neural tube defect by fetoscopic surgery
NCT03513757	An Observer-Blinded Randomized Study of Propofol Infusion vs Bolus Dexmedetomidine and Propofol Sedation for Pediatric Magnetic Resonance Imaging	Pediatric hydrocephalus	USA		The efficiency of propofol dexmedetomidine sedation compared with propofol infusion
NCT00670735	HCRN Core Data Project: Characterizing Patient Populations in the Hydrocephalus Clinical Research Network (HCRN)	Pediatric hydrocephalus	USA, Canada		To describe the number and characteristics of neurosurgical hydrocephalus patient events to HCRN Clinical Centers such as patient demographics, etiology of hydrocephalus, diagnostic information, as well as surgical and medical management decisions
NCT02601339	Beside Monitor of Cerebral Metabolism in Premature Infants With Intraventricular Hemorrhage and Post-Hemorrhagic Hydrocephalus	Post-hemorrhagic hydrocephalus	USA		Cerebral oxygen metabolism (CMRO2)
